# Supplementary material for: Efficient hyperactive piggyBac transgenesis in Plodia pantry moths
Source: Front Genome Ed. 2022 Dec 23;4:1074888. doi: 10.3389/fgeed.2022.1074888 (PMC9816379; doi:10.3389/fgeed.2022.1074888)
Supplement: Supplementary file 1 [file DataSheet1.PDF]

TABLE S1 | Mapping 5' *PiggyBac* insertions in the *Plodia interpunctella* genome using Splinkerette PCR.

***Plodia* line**	**Restriction Enzyme**	**Sequencing primer** Potter *et al.* 2010	**NCBI *Plodia interpunctella* reference genome**	**Contig/Scaffold**	**Position (>99% match)**	**Sequence** **Orange: *pBac* end** **Black : *Plodia* genome**
Pi_wFog [3xP3::DsRed]	BfuCI	5'SPLNK PB-SEQ	*plodia_v1*<br>GenBank: GCA_001368715.1	scaffold49<br>LN813179.1	151,615 - 152,193 (+/+)	TTTAGAAGAGAGAGCAATATTTCNAGAATGCATGCGTCAATTT<br>TACGCAGACTATCTTTCTAGGGTTAATCACACAGATTAAGAAA<br>AAGGGTGTACGTAATGTGACAGGTCGATGCATCTGCCTTGTGG<br>TCAATTTGTGTATTTA<br>GAGTTCGTATCCTTTCATTAAAACTTTGAGTTCTCTTTAGTTA<br>TTTATAGGGGTAATATCAAGGGTAAAACTCTCTTTAAATTGCT<br>AAAGTATTTCATTAAATAATGTTTTTTTAACTACTGAGCGAAC<br>AGGCATACGGTCCACCTGATGGGATGTGGTT<br>ACCGCTGCCTATGGATGACTGCAACACCAACGGTGTCACACGC<br>GCGTTACCAGCCCTGATTAAATCTATTCACGCCCTTTTTGAAG<br>TATACGTGTAGTCTCTAGCGGAAACCACGTTTTTCTAGGGAAA<br>AGTAGGAAAGCGGACTCTAGGCTCCGACGCT<br>CAACGGCTGAGGAAGAAAGGAGCCGTGAAAACGATAAGATGCC<br>GTCAAGAATATACGTGTCCATGGTCTAACAATTTTGGATGTAA<br>AGTTGGCTAAAAGTAGGAGTGTTGAAAAATCCTGGACTCCCCC<br>ACTCCAAGGCCGAGAAGCAAGCGAAAGCATT<br>CAGATAACACACAGAGATCCCACTAGTGTCGACACC
Pi_wFog [3xP3::EYFP; attP]	BfuCI	5'SPLNK PB-SEQ	*plodia_v1*<br>GenBank: GCA_001368715.1	contig_1640<br>CELO01001640.1	254,318 - 255,009 (+/+)	TTTAGAAGAGAGAGCAATATTTCAAGAATGCATGCGTCAATTT<br>TACGCAGACTATCTTTCTAGGGTTAAATGGTTTCGTTATACAT<br>GTCACACACGCATTTTAACTTATTTTCGAAAAAATATTTTCAT<br>AAGTAATATAAGTA<br>GTTCTCTATTGGACACAGTTGCCACAATTTACTAAACGGAAAA<br>TCAATCTGTGGCGATTAAAGTCCGTTTAATAAAGTTAAAACGA<br>AAATACTACTTTTTAATGCTGTCCCAGAATTAGTCCCAGACAG<br>ATGTTTAAAATTTGCCTCTGTTTAAAAGAAG<br>TTGATAATATATAATATGTATATATAGGTACTCCTACTCACTC<br>ATCTATACTATTATTATAAAGAGGTAAGCGTTTGTGAGTTTGT<br>ACGTTTGAAGCGGGCAATCTTCGAAACTACCGAACCGATTTCA<br>AAAATTCACTATTAGAAAGGTACATTGTCCA<br>AGATTGCTATAGGCTATATTTTATCTCATAATTCCCACGGGAG<br>CGAAGCCCCGGGCAACATCTAGTAAGTTCATAATTCCATTTTT<br>AAGTTTACTTACCTACACAGTCTTCTTTCTGTACTCTATTCTA<br>CTCCAAAGGCATTGAACAGTAAAAACATACC<br>TTGAAAGGTCTTAAATAAACATCGTAACCAATTGAATCGTATC<br>GTAATACTGTAGTATTACGATAAGCAAACGACCTCGGTGGCGC<br>AGTGGTAAAGTGCTTGCCTCTGAACCGTGAGGTCNCGGGTTCG<br>ATCCCACTAGTGTCGACACCAGTCTCA
