# Supplementary material for: Efficient hyperactive piggyBac transgenesis in Plodia pantry moths
Source: Front Genome Ed. 2022 Dec 23;4:1074888. doi: 10.3389/fgeed.2022.1074888 (PMC9816379; doi:10.3389/fgeed.2022.1074888)
Supplement: Supplementary file 2 [file Image1.PDF]

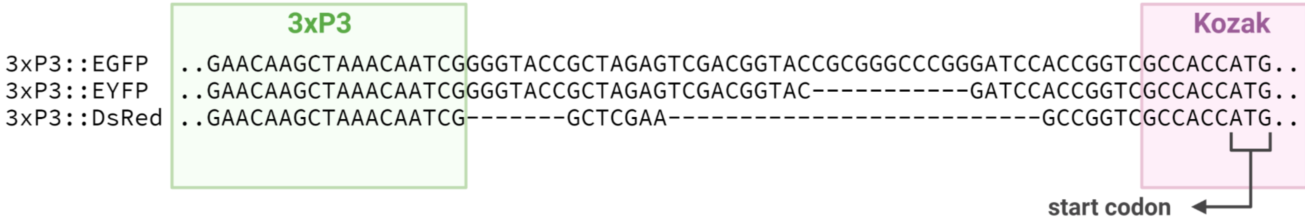

FIGURE S1 | Alignment of the region spanning, from left to right, the 3' end of the *3xP3* promoter and Kozak sequence preceding the fluorophore genes in 3 *pBac* donor plasmids.
